# Clinical implications of insulin-like growth factor II mRNA-binding protein 3 expression in non-small cell lung carcinoma

**DOI:** 10.3892/ol.2015.2910

**Published:** 2015-01-27

**Authors:** JINHUI ZHANG, YINGFU OU, YIBING MA, LINLIN ZHENG, XIAOKANG ZHANG, RONGJUN XIA, FANYONG KONG, YUE SHEN, SHIQING WANG, LIJUAN LIN

**Affiliations:** 1 Institute of Molecular Medicine, Medical College, Eastern Liaoning University, Dandong, Liaoning 118000, P.R. China; 2 Department of Pathology, Dandong Centre Hospital, Dandong, Liaoning 118000, P.R. China

**Keywords:** insulin-like growth factor II mRNA-binding protein 3, non-small cell lung carcinoma, prognosis, survival analysis

## Abstract

In order to examine the role of insulin-like growth factor II mRNA-binding protein 3 (IMP3) expression for the prognostic evaluation of non-small cell lung carcinoma (NSCLC), a total of 186 breast cancer patients, with adjacent non-tumor lung tissues, were selected for immunohistochemical staining of IMP3 protein. The NSCLC tissues and paired adjacent non-tumor tissues of six patients were quantified using reverse transcription quantitative polymerase chain reaction. The correlations between IMP3 overexpression and the clinical features of NSCLC were evaluated using the χ^2^ test and Fisher’s exact test. The survival rate was calculated using the Kaplan-Meier method, and the association between prognostic factors and patient survival was also analyzed by Cox’s proportional hazards models. The results showed that IMP3 protein exhibited a mainly cytoplasmic staining pattern in the NSCLC tissues. The positive rate of IMP3 protein expression was 74.7% (139/186) in the NSCLC tissues and was significantly higher than the rate of 19.9% (37/186) in the adjacent non-tumor tissues. The expression rate of the NQO1 protein was correlated with a large tumor size, poor differentiation, lymph node metastasis, late clinical stage, and disease-free and overall survival rates in the NSCLC patients. In the early- and late-stage NSCLC groups, the disease-free and overall survival rates of the patients with IMP3 expression were significantly lower than those of the patients without IMP3 expression. Further analysis using Cox’s proportional hazard regression model revealed that IMP3 expression was a significant independent hazard factor for the overall survival rate of patients with NSCLC. In conclusion, the present study found that IMP3 plays a significant role in the progression of NSCLC, and that it may potentially be used as an independent biomarker for prognostic evaluation of the cancer.

## Introduction

Lung cancer is divided into non-small cell lung cancer (NSCLC) and SCLC according to its morphology. Accounting for ~80% of all lung cancers, NSCLC is clinically a heterogeneous entity with major histological subtypes, including adenocarcinoma, squamous cell carcinoma and large cell carcinoma (1). Slower rates of growth and spread compared with SCLC are common features of all NSCLC subtypes. This enables early eradication of the cancer by surgery, however, only a small fraction of cases are currently diagnosed in clinical stages I-IIb, where surgical removal is the preferred therapeutic option (2). Therefore, there is a requirement for more sensitive NSCLC biomarkers, which can predict prognosis and guide the effective targeted therapy.

The oncofetal protein, insulin-like growth factor II mRNA-binding protein 3 (IMP3), is a member of the IMP family that has recently become a focus of attention, as it appears to be significant in the migration and adhesion of cells in range of malignant neoplasms (3). IMP3 is a 580-amino acid protein, with four K homology domains and two RNA recognition motifs, and is encoded by a gene on chromosome 7p11.5 (4) that has been known in previous studies as K homology domain-containing protein overexpressed in cancer or L523S. L523S is a regulatory binding protein that is believed to be involved in the facilitation of insulin-like growth factor (IGF)-II production by stabilizing and trafficking it intracellularly (5). IMP3 is expressed in numerous cells of the developing fetus, but not in the majority of adult cells, with the exception of the gonads. IMP3 overexpression has been observed in a number of malignant tumors, including renal carcinomas (6), malignant pancreatic lesions (7), endometrial carcinomas (8), and uterine cervical (9) and testicular (10) cancer.

Our previous studies showed that that IMP3 expression is correlated with the prognosis of a variety of human tumors, such as hepatocellular carcinoma and colorectal cancer (11,12). Thus, IMP3 is expected to be a novel molecular target for cancer therapy. However, the role of IMP3 in prognostic evaluation and its association with survival in NSCLC is unknown. As IMP3 is known to play a critical role in numerous cancers, the present study analyzed its function in NSCLC.

## Materials and methods

### Clinical samples

Fresh samples from eight cases of NSCLC were paired with adjacent non-cancerous tissues, and 186 NSCLC cases with routinely processed and paraffin-embedded tissue samples, which met the strict follow-up criteria, were selected at random from patients undergoing surgery between 2004 and 2008 at the Dandong Centre Hospital (Dandong, Liaoning, China). Pathological parameters, including age, gender, smoking status, tumor size, pathological stage, differentiation, subtype, CEA level, metastasis status, and disease-free and overall survival data, were carefully reviewed. The ages of the patients ranged between 32 and 79 years, with a mean age of 62.4 years. The male to female ratio was 112:74. Tumors were staged according to the 6th edition of the American Joint Committee on Cancer (13). Of the 186 NSCLC samples, 97 were determined to be early stage (I-II) and 89 were late stage (III-IV). A total of 43 samples were well-differentiated cancer, 87 were moderately-differentiated cancer and 56 were poorly-differentiated cancer. No patients received chemotherapy or radiotherapy prior to surgery. By March 2013, 66 patients had succumbed and 120 patients remained alive. The median survival time was 69 months. This study was approved by the Ethics Committee of Eastern Liaoning University (Dandong, China) and written informed consent was obtained from all patients.

### RNA extraction and reverse transcription quantitative polymerase chain reaction (RT-qPCR)

Total RNA (2 μl) from fresh tissues was extracted using TRIzol reagent (Takara Biotechnology, Dalian, China). First-strand cDNA was synthesized using PrimeScript reverse transcriptase (Takara Biotechnology) and oligo(dT) following the manufacturer’s instructions. All PCR reactions were performed in a 20 μl reaction mixture (10× PCR Buffer II 2 μl, dNTP mixture (2.5 mM each) 1.6 μl, primer-forward 1 μl, primer-reverse 1 μl, Takara Ex Taq HS (5 U/μl) 0.15 μl, cDNA 1 μl and RNAse Free <20 μl). Firstly, cDNA was denatured for 4 min at 94°C. Next, PCR amplification was performed for 23–36 cycles at 94°C for 15 sec and 53–58°C for 30 sec to anneal the primers, with a final extension step at 72°C for 1 min. Aliquots (10 μl) of the PCR reaction mixture were removed after 6, 9, 12 and 15 cycles and separated by electrophoresis on 3% agarose gels. Images were captured using the Champchemi Professional image analysis system (Sagecreation, Beijing, China), and quantitation was performed using LANE 1D software (Sagecreation). To examine expression, qPCR was performed with a Bio-Rad sequence detection system (Hercules, CA, USA), according to the manufacturer’s instructions, using a double-stranded DNA-specific SYBR Premix Ex Taq™ II kit (Takara Biotechnology). Double-stranded DNA-specific expression was tested by the comparative Ct method using 2-ΔΔCt. The primers were as follows: IMP3 forward, 5′-CCTTTGCTGCTGGCAGAGTT-3′ and reverse, 5′-AACAAAGGGAAGTGCAGAGC-3′; and GAPDH forward, 5′-GGTCTCCTCTGACTTCAACA-3′ and reverse, 5′-ATACCAGGAAATGAGCTTGA-3′. All assays were performed in triplicate and repeated at least three times.

### Immunohistochemical analysis

For immunohistochemical study using the DAKO Labeled Streptavidin Biotin kit (DAKO, Glostrup, Denmark), 4-μm thick tissue sections were deparaffinized, rehydrated and incubated with 3% H_2_O_2_ in methanol for 15 min at room temperature to eliminate endogenous peroxidase activity. The antigen was retrieved at 95°C for 20 min by placing the slides in 0.01 M sodium citrate buffer (pH 6.0). The slides were then incubated with the polyclonal goat anti-IMP3 antiserum (1:150; N-19; Santa Cruz Biotechnology, Santa Cruz, CA, USA) and monoclonal mouse anti-Ki-67 antiserum (MAB-0129; Maixin Technology Co., Ltd., Shenzen, Guangdong, China) primary antibodies at 4°C overnight. Following incubation at room temperature for 30 min with biotinylated secondary antibodies; rabbit anti-goat and goat anti-mouse (dilution, 1:200; ZSGB Biotechnology Co., Ltd., Beijing, China), the slides were incubated with streptavidin-peroxidase complex at room temperature for 30 min. Immunostaining was developed by using 3,3′-diaminobenzidine as a chromogen and then counterstained with Mayer’s hematoxylin. Goat immunoglobulin G isotope controls were used, which showed negative staining. Additionally, the positive tissue sections were processed without the primary antibody to create the negative controls.

All specimens were examined by two pathologists who were blinded to the clinical data. In cases of discrepancy, a final score was established when an agreement was reached following reassessment of the samples under a double-headed microscope. Briefly, immunostaining for IMP3 was semi-quantitatively scored as follows: −, 0 to <5% positive cells; +, 5–50% positive cells; and ++, >50% positive cells. Only the cytoplasmic expression pattern was considered as positive staining. For the survival analysis, IMP3 expression levels were denoted as either positive (+ and ++) or negative (−) expression.

### Statistical analyses

Statistical analyses were performed using SPSS software, version 17.0 (SPSS, Inc., Chicago, IL, USA). Correlations between IMP3 expression and clinicopathological characteristics were evaluated using the χ^2^ test and Fisher’s exact test. The disease-free and overall survival rates following tumor removal were calculated using the Kaplan-Meier method, and differences in survival curves were analyzed using log-rank tests. Multivariate survival analysis was performed on all significant characteristics measured by univariate survival analysis with Cox’s proportional hazard regression model. P<0.05 was considered to indicate a statistically significant difference.

## Results

### IMP3 protein expression in NSCLC samples

**Figure 1:**
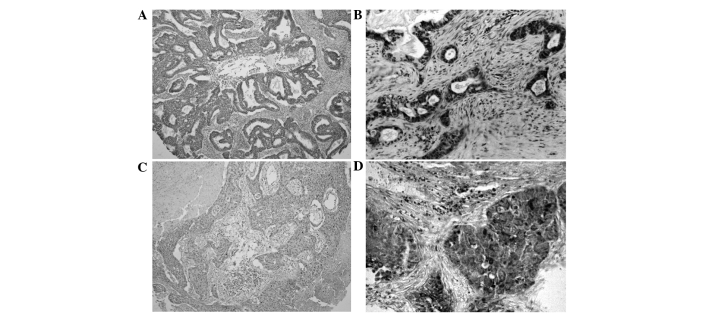
Immunohistochemical staining of insulin-like growth factor II mRNA-binding protein 3 in non-small cell lung cancer tissues. (A) Negative (magnification, ×50) and (B) positive staining in lung adenocarcinoma (magnification, ×200); and (C) negative (magnification, ×50) and (D) positive staining in lung squamous cell carcinoma (magnification, ×200).

IMP3 protein expression exhibited a strict nuclear staining pattern in the NSCLC tissues upon immunohistochemistry analysis, with the exception of three cases of adenocarcinoma, which showed a mainly cytoplasmic staining pattern. IMP3 protein expression was negative in the normal lung tissues, but was usually upregulated in the NSCLC tissues. The positive rate of IMP3 protein was 74.7% (139/186) in the NSCLC tissues, and was significantly higher than the 19.9% (37/186) found in the normal lung tissues (P<0.01) (Fig. 1; Table I).

**Figure 2:**
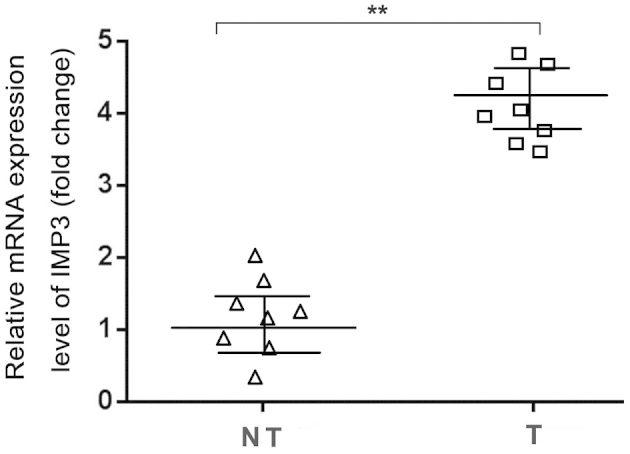
Reverse transcription quantitative polymerase chain reaction analysis of insulin-like growth factor II mRNA-binding protein 3 (IMP3) mRNA expression in eight cases of fresh NSCLC (T) and non-tumor tissue (NT) samples. Experiments were performed in triplicate for each case. IMP3 mRNA expression levels were significantly higher in the NSCLC samples compared with the adjacent non-tumor tissues (^**^P<0.01). NSCLC, non-small cell lung cancer.

The RT-qPCR data confirmed increased levels of IMP3 mRNA expression in the NSCLC samples compared with the adjacent non-tumor tissues (Fig. 2).

### Correlation between IMP3 expression and the clinicopathological features of NSCLC

To evaluate the role of IMP3 protein in NSCLC progression, the correlations between IMP3 protein expression and the major clinicopathological features of NSCLC were analyzed. The results showed that IMP3 expression was significantly associated with tumor size, differentiation, lymph node metastasis and the clinical stage of NSCLC (P=0.013, P<0.001, P=0.004 and P<0.001, respectively). However, IMP3 expression levels were not associated with age, gender, CEA level, smoking status or pathological subtype of NSCLC (P>0.05) (Table II).

### Correlation between survival rates and IMP3 expression using the Kaplan-Meier method

**Figure 3:**
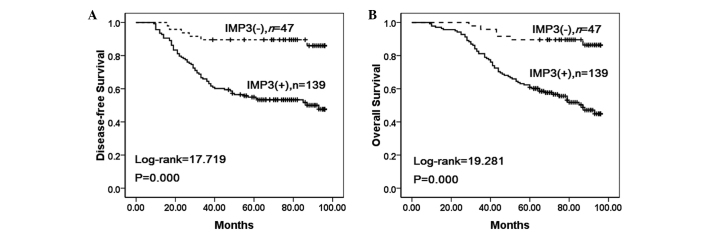
Kaplan-Meier analysis of disease-free and overall survival rates in 196 non-small cell lung cancer (NSCLC) patients in relation to IMP3 protein expression. (A) NSCLC patients with insulin-like growth factor II mRNA binding protein 3 (IMP3)-positive expression exhibited a lower disease-free survival rate compared with those without IMP3 expression. (B) NSCLC patients with IMP3-positive expression exhibited a lower overall survival rate compared with those without IMP3 expression.

To further confirm the role of IMP3 expression in NSCLC progression, the disease-free and overall survival rates of 186 patients with NSCLC were analyzed using the Kaplan-Meier method. It was found that the NSCLC patients with IMP3 expression exhibited lower disease-free (log-rank=17.719, P<0.001) and overall (log-rank=19.281, P<0.001) survival rates compared with those patients without IMP3 expression (Fig. 3).

**Figure 4:**
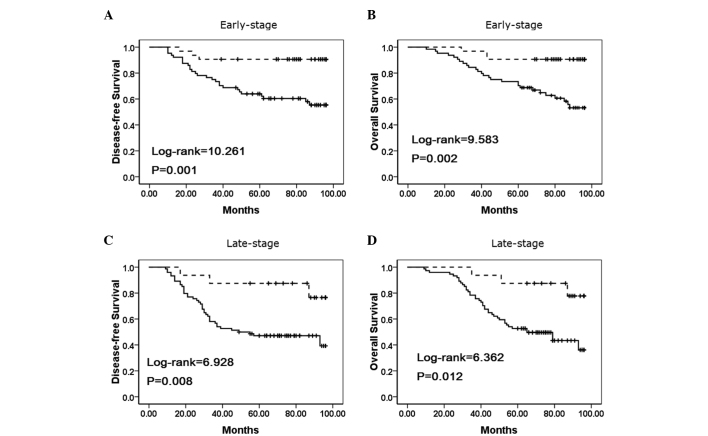
Kaplan-Meier analysis of disease-free and overall survival rates in 196 NSCLC patients with or without IMP3 expression in relation to clinical stage. (A) In the early stage, the disease-free survival rate of patients with IMP3-positive expression was lower than those without IMP3 expression. (B) In the early stage, the overall survival rate of patients with IMP3-positive expression was lower than those without IMP3 expression. (C) In the late stage, the disease-free survival rate of patients correlated with IMP3 expression. (D) In the late-stage, the overall survival rate of patients was correlated with IMP3 expression.

To substantiate the significance of IMP3 expression in NSCLC progression, the correlations between IMP3 expression and the clinical stage of NSCLC were analyzed. In early-stage NSCLC, the patients with IMP3 expression exhibited lower disease-free and overall survival rates compared with the patients without IMP3 expression (P=0.001 and P=0.002, respectively) (Fig. 4A–B). Additionally, the disease-free and overall survival rates were also correlated with IMP3 expression status (P=0.008, and P=0.012, respectively) in late-stage NSCLC (Fig. 4C–D).

### IMP3 is an independent prognostic factor in NSCLC, as determined by Cox’s proportional hazard regression model

Univariate analysis showed that the patients with NSCLC tumors that expressed IMP3 exhibited significantly lower overall survival rates (P<0.001) compared with the patients with NSCLC tumors that did not express IMP3. Additionally, patient age (P=0.024), pathological stage (P<0.001), differentiation (P=0.029), and lymph node metastasis (P=0.002) were all associated with the overall survival rate. Therefore, multivariate survival analysis was performed using Cox’s proportional hazards model for all the significant variables found with the univariate survival analysis. The results suggested that clinical stage [hazard ratio (HR), 1.734; 95% confidence interval (CI), 1.279–2.351; P<0.001), and lymph node metastasis (HR, 1.431; 95% CI, 1.054–1.944; P=0.022) were independent prognostic factors for overall survival rates in NSCLC. Significantly, IMP3 expression also emerged as a significant independent prognostic factor in the prognosis of NSCLC (HR, 1.608; 95% CI, 1.134–2.281; P=0.008) (Table III).

## Discussion

IMP3 is considered as the overexpressed K homology protein in carcinoma and also the activator of IGF-II mRNA translation. IGF-II, an embryonic growth factor, is structurally homologous to proinsulin and has also been found to be one of the endogenous genes. IGF-II plays a significant role in embryonic development and cell growth, and can promote cell proliferation and inhibit apoptosis (14). The expression of IGF-II is affected by various factors and its transcript contains six types of mRNA (15,16). IMP3 is mainly combined with IGF-II leader 3 mRNA, which can increase the expression of IFG-II by promoting the IFG-II leader 3 mRNA and exert carcinogenic effects through patterns decided by IGF-II. IMP3 promotes the proliferation of tumor cells by increasing the translation of IGF-II mRNA (14,17). IMP3 has been shown to be essential to cell adhesion and spread (14). The decreased expression of IMP1 and IMP3 is associated with the downregulation of mRNA, which encodes the extracellular matrix (ECM) and adhesive protein. The study by Vikesaa *et al* agreed that IMP3 can regulate the ECM and expression of particular adhesion proteins (such as ALCAM). IMP3 can also stabilize cluster of differentiation 44 mRNA and promote pseudopod structure formation in cancer cells, i.e., IMP3 acts like an oncogene (18).

The effect of IMP3 on tumors has become a focus of attention. Recent studies have shown that IMP3 is associated with the occurrence and development of several carcinomas. Yamamoto *et al* suggested that IMP3 may be an supplementary tool for the identification of aggressive abdominal mesenchymal tumors other than gastrointestinal mesenchymal tumors (19). Lee *et al* (20) suggested an independent association between IMP3 expression and disease recurrence, cancer-specific mortality and all-cause mortality in upper urinary tract urothelial carcinoma. This may aid in improving the risk stratification and prognostication of upper urinary tract urothelial carcinoma patients treated with radical nephroureterectomy (20). Beljan Perak *et al* (21) analyzed 105 patients with advanced lung adenocarcinoma by indirect enzyme immunohistochemistry, and found that IMP3 expression is associated with a solid subtype and with distant metastases, regardless of the histological subtype of the lung adenocarcinoma.

In our previous study, it was shown that IMP3 expression predicts a poor prognosis in patients with lung squamous cell carcinoma (22). The present study examined IMP3 expression and the clinicopathological features of NSCLC, and found that IMP3 expression was significantly correlated with a large tumor size, poor differentiation, positive node status and advanced clinical stage, but not with age, gender, pathological subtype, CEA level or smoking status of patients with NSCLC. With regard to survival, it was found that NSCLC patients with IMP3 expression exhibited lower disease-free and overall survival rates compared with patients without IMP3 expression. In either early- or late-stage NSCLC, patients with IMP3 expression exhibited lower disease-free and overall survival rates compared with those without IMP3 expression. Moreover, multivariate survival analysis demonstrated that IMP3 expression emerged as a significantly independent hazard factor for overall survival in NSCLC, along with clinical stage and metastasis. In conclusion, IMP3 plays an significant role in NSCLC progression and may be an independent biomarker for evaluating prognosis in patients with NSCLC.

## Figures and Tables

**Table I:** Insulin-like growth factor II mRNA-binding protein 3 protein expression in NSCLC.

		IMP3 protein expression, n				
				
Diagnosis	No. of cases	−	+	++	Positive rate, %	P-value
NSCLC	186	47	60	79	74.7	0.000^a^
Adjacent non-tumor	186	149	22	15	19.9	

a P<0.01 vs. adjacent non-tumor tissues.

NSCLC, non-small cell lung cancer.

**Table II:** Correlation between IMP3 expression and the clinicopathological features of non-small cell lung cancer.

	IMP3 protein expression, n (%)			
			
Variables	+/++	−	χ^2^	P-value
Age, years			0.458	0.500
<62	66 (72.5)	25 (27.5)		
≥62	73 (76.8)	22 (23.2)		
Gender			2.624	0.106
Male	79 (70.5)	33 (29.5)		
Female	60 (81.1)	14 (18.9)		
Tumor size, cm			6.214	0.013^a^
≤3	51 (65.4)	27 (34.6)		
>3	88 (81.5)	20 (18.5)		
Differentiation			19.170	0.000^b^
Well	22 (51.2)	21 (48.8)		
Moderately	67 (77.0)	20 (23.0)		
Poorly	50 (89.3)	6 (10.7)		
Pathological subtype			1.018	0.314
SCC	68 (78.2)	19 (21.8)		
AC	71 (71.7)	28 (28.3)		
Clinical stage			20.761	0.000^b^
I–II	59 (60.8)	38 (39.2)		
III–IV	80 (89.9)	9 (10.1)		
LN metastasis			8.331	0.004^b^
Positive	84 (83.2)	17 (16.8)		
Negative	55 (64.7)	30 (35.3)		
CEA level			0.037	0.849
Normal	54 (74.0)	19 (26.0)		
Increased	85 (75.2)	28 (24.8)		
Smoking status			0.364	0.547
Yes	94 (73.4)	34 (26.6)		
No	45 (77.6)	13 (22.4)		

Statistical analyses were performed using Pearson χ^2^ tests.

a P<0.05 and

b P<0.01.

IMP3, insulin-like growth factor II mRNA-binding protein 3; SCC, squamous cell carcinoma; AC, adenocarcinoma; CEA, carcinoembryonic antigen.

**Table III:** Univariate and multivariate survival analysis of clinicopathological factors for the overall survival rate of 186 patients with non-small cell lung cancer.

					95% CI		
						
Characteristics	β	SE	Wald	HR	Lower	Upper	P-value
Univariate							
Gender	0.100	0.148	0.459	1.105	0.827	1.477	0.498
Age, years	0.349	0.155	5.071	1.418	1.046	1.921	0.024^a^
Smoking status	0.103	0.177	0.340	1.109	0.784	1.569	0.560
Tumor size, cm	0.242	0.149	2.634	1.274	0.951	1.708	0.105
Clinical stage	0.588	0.151	15.198	1.801	1.340	2.420	0.000^b^
Differentiation	0.229	0.105	4.746	1.257	1.023	1.544	0.029^a^
CEA	0.023	0.147	0.024	1.023	0.767	1.365	0.876
Pathological subtype	0.050	0.147	0.116	1.051	0.788	1.404	0.734
LN metastasis	0.478	0.151	10.029	1.614	1.200	2.170	0.002^b^
IMP3	0.618	0.169	13.382	1.856	1.333	2.585	0.000^b^
Multivariate							
Age, years	0.283	0.157	3.251	1.328	0.976	1.806	0.071
Clinical stage	0.551	0.155	12.583	1.734	1.279	2.351	0.000^b^
Differentiation	0.137	0.112	1.511	1.147	0.922	1.428	0.219
LN metastasis	0.359	0.156	5.277	1.431	1.054	1.944	0.022^a^
IMP3	0.475	0.178	7.096	1.608	1.134	2.281	0.008^b^

Statistical analyses were performed using Cox’s proportional hazard regression model.

a P<0.05 and

b P<0.01.

CI, confidence interval; HR, hazard ratio; SE, standard error; CEA, carcinoembryonic antigen; LN, lymph node.

## References

[1] Brambilla E, Travis WD, Colby TV (2001). The new World Health Organization classification of lung tumours. Eur Respir J.

[2] Lokk K, Vooder T, Kolde R (2012). Methylation markers of early-stage non-small cell lung cancer. PLoS One.

[3] Ikenberg K, Fritzsche FR, Zuerrer-Haerdi U (2010). Insulin-like growth factor II mRNA binding protein 3 (IMP3) is overexpressed in prostate cancer and correlates with higher Gleason scores. BMC Cancer.

[4] Mentrikoski MJ, Ma L, Pryor JG (2009). Diagnostic utility of IMP3 in segregating metastatic melanoma from benign nevi in lymph nodes. Mod Pathol.

[5] Hoffmann NE, Sheinin Y, Lohse CM (2008). External validation of IMP3 expression as an independent prognostic marker for metastatic progression and death for patients with clear cell renal cell carcinoma. Cancer.

[6] Jiang Z, Chu PG, Woda BA (2008). Combination of quantitative IMP3 and tumor stage: a new system to predict metastasis for patients with localized renal cell carcinomas. Clin Cancer Res.

[7] Schaeffer DF, Owen DR, Lim HJ (2010). Insulin-like growth factor 2 mRNA binding protein 3 (IGF2BP3) overexpression in pancreatic ductal adenocarcinoma correlates with poor survival. BMC Cancer.

[8] Mhawech-Fauceglia P, Herrmann FR, Rai H (2010). IMP3 distinguishes uterine serous carcinoma from endometrial endometrioid adenocarcinoma. Am J Clin Pathol.

[9] Lu D, Yang X, Jiang NY (2011). IMP3, a new biomarker to predict progression of cervical intraepithelial neoplasia into invasive cancer. Am J Surg Pathol.

[10] Hammer NA, Hansen Tv, Byskov AG (2005). Expression of IGF-II mRNA-binding proteins (IMPs) in gonads and testicular cancer. Reproduction.

[11] Chen LT, Lin LJ, Zheng LL (2013). The correlation between insulin-like growth factor II mRNA binding protein 3 expression in hepatocellular carcinoma and prognosis. Hepatogastroenterology.

[12] Lin L, Zhang J, Wang Y (2013). Insulin-like growth factor-II mRNA-binding protein 3 predicts a poor prognosis for colorectal adenocarcinoma. Oncol Lett.

[13] Singletary SE, Greene FL, Sobin LH (2003). Classification of isolated tumor cells: clarification of the 6th edition of the American Joint Committee on Cancer Staging Manual. Cancer.

[14] Liao B, Hu Y, Brewer G (2011). RNA-binding protein insulin-like growth factor mRNA-binding protein 3 (IMP-3) promotes cell survival via insulin-like growth factor II signaling after ionizing radiation. J Biol Chem.

[15] Gredes T, Spassov A, Mai R (2009). Changes in insulin like growth factors, myostatin and vascular endothelial growth factor in rat musculus latissimus dorsi by poly-3-hydroxybutyrate implants. J Physiol Pharmacol.

[16] Ozdemir NO, Türk NS, Düzcan E (2011). IMP3 expression in urothelial carcinomas of the urinary bladder. Turk Patoloji Derg.

[17] Liao B, Hu Y, Herrick DJ, Brewer G (2005). The RNA-binding protein IMP-3 is a translational activator of insulin-like growth factor II leader-3 mRNA during proliferation of human K562 leukemia cells. J Biol Chem.

[18] Vikesaa J, Hansen TV, Jønson L (2006). RNA-binding IMPs promote cell adhesion and invadopodia formation. EMBO J.

[19] Yamamoto H, Arakaki K, Morimatsu K (2014). Insulin-like growth factor II messenger RNA-binding protein 3 expression in gastrointestinal mesenchymal tumors. Hum Pathol.

[20] Lee DJ, Xylinas E, Rieken M (2014). Insulin-like growth factor messenger RNA-binding protein 3 expression helps prognostication in patients with upper tract urothelial carcinoma. Eur Urol.

[21] Beljan Perak R, Durdov MG, Capkun V (2012). IMP3 can predict aggressive behaviour of lung adenocarcinoma. Diagn Pathol.

[22] Lin L, Zhang J, Wang Y (2013). Expression of insulin-like growth factor 2 mRNA-binding protein 3 expression and analysis of prognosis in the patients with lung squamous cell carcinoma. Xi Bao Yu Fen Zi Mian Yi Xue Za Zhi.

